# Social phenotype-dependent selection of social environment in wild great and blue tits: an experimental study

**DOI:** 10.1098/rspb.2022.1602

**Published:** 2022-11-09

**Authors:** Charlotte E. Regan, Kristina B. Beck, Keith McMahon, Sam Crofts, Josh A. Firth, Ben C. Sheldon

**Affiliations:** Edward Grey Institute, Department of Biology, University of Oxford, 11a Mansfield Road, Oxford OX1 3RT, UK

**Keywords:** blue tit, density, great tit, matching habitat choice, social environment, social phenotype

## Abstract

There is growing evidence that individuals actively assess the match between their phenotype and their environment when making habitat choice decisions (so-called matching habitat choice). However, to our knowledge, no studies have considered how the social environment may interact with social phenotype in determining habitat choice, despite habitat choice being an inherently social process and growing evidence for individual variation in sociability. We conducted an experiment using wild great and blue tits to understand how birds integrate their social phenotype and social environment when choosing where and how to feed. We used programmable feeders to (i) record social interactions and estimate social phenotype, and (ii) experimentally manipulate the local density experienced by birds of differing social phenotype. By tracking feeder usage, we estimated how social environment and social phenotype predicted feeder choice and feeding behaviour. Both social environment and social phenotype predicted feeder usage, but a bird's decision to remain in a particular social environment did not depend on their social phenotype. By contrast, for feeding behaviour, responses to the social environment depended on social phenotype. Our results provide rare evidence of matching habitat choice and shed light on the dependence of habitat choice on between-individual differences in social phenotype.

## 1. Introduction

Understanding variation in animal habitat selection behaviour is a central aim within ecology. This is owing to the critical role that habitat selection plays in connecting environmental heterogeneity to individual fitness, and thus in driving population dynamics and evolutionary processes [1,2]. Studies attempting to explain pronounced between-individual differences in habitat choice have revealed that such variability can be explained by differences in the environment experienced by individuals [3], phenotypic differences between them [4], and habitat preference genes [5]. Characteristics such as age [6,7], sex [8,9] and body size [10,11] have all been linked to variability in habitat selection behaviour, probably owing to their effects on individual condition/internal state and therefore on the relative costs/benefits of making a given movement [12].

The effects of environmental heterogeneity on fitness may differ between individuals with different phenotypes. As such, individuals' phenotypes may also directly shape habitat selection through interactions with environmental variability. Therefore, rather than altering their phenotype to better suit environmental conditions (i.e. phenotypic plasticity), individuals may opt to relocate in order to find an environment that is better suited to their phenotype [13]. This process, whereby individuals actively assess the match between their phenotype and a specific habitat to maximize their fitness, was termed ‘matching habitat choice’ by Edelaar *et al.* [13]. It is of substantial interest because it can lead to the spatial clustering of individuals with similar phenotypes, and thereby generate correlations between phenotype and environment, and thus contribute to maintaining phenotypic variation within populations and facilitate local adaptation [13–16].

Despite the potential population-level consequences of matching habitat choice, and the fact it has been introduced on many occasions under various terms (summarized in [13]), it has, until recently, received little attention within ecological and evolutionary research. Fortunately, there is now a growing number of empirical and theoretical studies testing for the occurrence of matching habitat choice (e.g. [17–19]), exploring the conditions under which it may occur [19,20], and understanding the consequences of matching habitat choice for populations now and into the future as human-driven environmental change continues [15,21–23]. To date, much of the empirical work testing for the presence of matching habitat choice has focused on morphological traits, such as body size or shape [19,24], bill length [25,26] and plumage or body colour [16,17,27], owing to the expected link between such traits and habitat characteristics given their importance for obtaining food resources or avoiding predation. However, other lesser studied traits, such as personality attributes, may also have a role to play in driving matching habitat choice [28].

Consistent between-individual differences in suites of behavioural traits (i.e. personality) may alter how individuals interact with their environment and therefore how the environment affects survival and reproduction. Although stable between-individual differences in personality have been widely demonstrated across species [29], and covariances between personality and environmental conditions having also been documented [30–32], the study of so-called ‘personality-matching habitat choice’ [28,31] is in its infancy.

Social phenotype is a particularly promising trait for study in the context of matching habitat choice because habitat choice is an inherently social process, with the social environment having the potential to both negatively (e.g. through competition and stress) and positively (e.g. social information transfer or reduction in predation risk) influence individual fitness. Thus, we might expect individuals to respond to the social environment when making habitat choice decisions, and if individual fitness depends on the interaction between their social phenotype and the social environment, then we may also expect individuals with different social phenotypes to select contrasting social environments. For example, asocial individuals may have reduced fitness at high density because they may experience higher stress at high density or may be subject to more aggression. Thus, they may prefer to move to areas of lower density. Though we already have evidence to suggest that the social environment plays a part in determining habitat selection behaviour [33–36], little consideration has been paid to the ways that social phenotype and social environment might interact to determine individual habitat choice (but see [37] and [38]). The lack of research in this area is probably owing to a two-fold challenge of collecting appropriate data. First, it is difficult to follow habitat-choice decisions in sufficiently large samples of wild individuals with known social phenotypes. Second, it is challenging to establish causal (rather than correlational) relationships between social phenotype and movement without manipulating the social environment.

In this study, we exploited a long-term large-scale study of individual great tits (*Parus major*) and blue tits (*Cyanistes* caeruleus) that is ideally suited for studying the social dependence of individual habitat choice. This is because a combination of long-term tagging of birds with passive integrated transponders and the development of programmable feeders with radio-frequency identification (RFID) antennae enables the characterization of individual social phenotype [39,40] and the manipulation of the social environment that birds are exposed to (e.g. [41,42]). Using this system, we aimed to understand (i) whether individuals with different social phenotypes selected different social environments (high or low local density), and (ii) if individuals with different social phenotypes adjusted their feeding behaviour in response to different social environments (high or low local density).

## 2. Material and methods

### (a) Study population

This study was conducted from December 2020 to March 2021 within the long-term individual-based study of great and blue tits in Wytham Woods, Oxfordshire, UK (51°46′ N, 1°20′ W). Within this population, birds are ringed either as nestlings, as breeding adults captured at nestboxes, or as immigrants via mist-netting during the autumn/winter. Upon the first capture, individuals are tagged with both unique British Trust for Ornithology metal leg rings and plastic leg rings containing a passive integrated transponder (PIT; Eccel Technology Ltd, Leicester, UK). Previous work in this system has estimated that the ringing protocol detailed above results in over 90% of birds in the population being PIT-tagged [39]. Using data from the breeding season following our experiment (i.e. 2021), we estimate that 80% of breeding birds were already tagged in the winter (note that this figure included untagged immigrants that joined the population after our experimental phase). Feeders fitted with RFID antennae (Nature Counters, Maidstone, UK) then record visits of individual birds to the feeder during the winter when tits form mixed-species foraging flocks that exhibit fission–fusion dynamics [43,44]. These data can be used to construct social networks and characterize individual social behaviour.

### (b) Pre-experimental period

**Figure 1.**
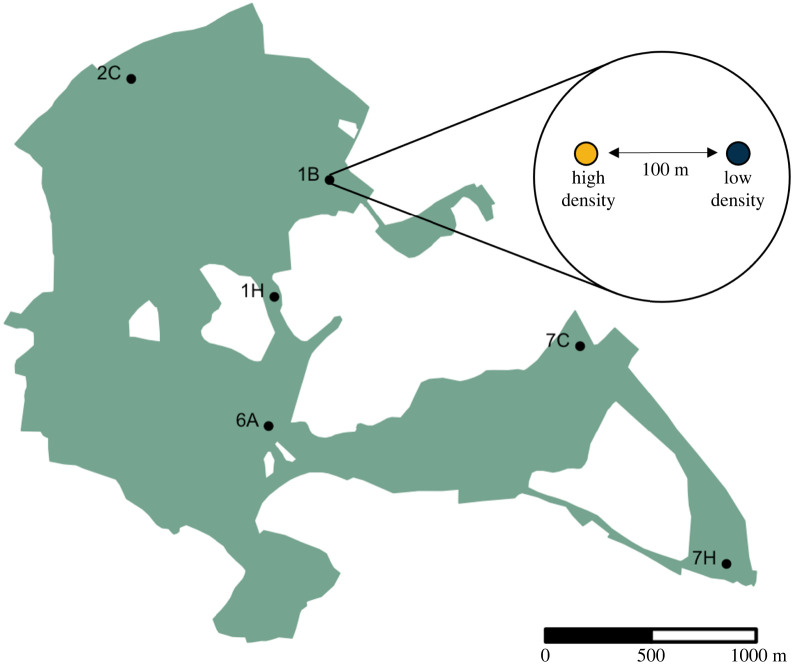
Six experimental sites were distributed across the woodland, with each site having two programmable RFID feeders separated by 100 m. The low-density feeder was programmed to allow access to a randomly selected 20% of local birds recorded in the pre-experimental phase, while the high-density feeder was programmed to allow access to all birds other than those given access to the low-density feeder. (Online version in colour.)

In the pre-experimental phase, we set up six experimental sites (figure 1), each with two RFID feeders 100 m apart (figure 1). Feeders were positioned within metal cages (see the electronic supplementary material, figure S1) to prevent squirrels damaging the feeders. This set-up allows birds to ‘queue’ in the vicinity of the feeder while waiting to access the feeder itself (see videos in the electronic supplementary material). Because of an initial equipment shortage, four sites went live in December (1B, 2C, 6A and 7C), while the remaining two (1H and 7H) went live in January. The two feeders at each site were programmed to allow all birds access 24 h per day during this time. Feeders were visited every two to three days, at which point data were downloaded, feeder performance was checked, and feeders were topped up with sunflower seeds. Prior to starting the experimental manipulations, we selected the most recent 12 to 16 days of feeder data for each site (variation caused by occasional short-term feeder malfunctions) to construct pre-experimental social networks and obtain measures of individual social phenotypes prior to implementing the manipulation. Experimental sites were separated by sufficient distance to make between-site movements during the course of the experiment unlikely. Indeed, of 8266 daily records, we only recorded 106 between-site movements over the course of the experimental period.

We used an established protocol to process the data downloaded from the feeders into an appropriate format for constructing social networks. First, we used a Gaussian mixture model (‘gmmevents’ function in the ‘asnipe’ package [45]) to identify gathering events (i.e. flocks) at each feeder on each day and thereby assign individuals to the flock they were most likely to have been a member of [46]. This approach provides an objective way of assigning group membership, producing networks that are more robust than those produced using alternative methods [47]. Furthermore, the associations derived using a Gaussian mixture model have been shown to reflect social ties rather than coincidental associations in this system, and the resulting networks replicate known biological phenomena, including the maintenance and development of social ties between existing and new breeding pairs [46]. Group membership information was then translated into a ‘group-by-individual’ matrix detailing co-occurrences of all birds during the pre-experimental period. We then used the ‘simple ratio index’ [48] to calculate weighted social associations between birds to create social networks for each experimental site. Once social networks had been constructed for each site, we calculated weighted degree centrality (i.e. the sum of all their edge weights to all their associates) for all individuals in the network as our measure of individual social phenotype. We chose weighted degree as it is an intuitive measure of individual sociability that is widely used in animal social network studies to assess individuals general sociability [49], and also this is a metric that has been shown to be repeatable in this system and act as a ‘social phenotype’ [39,40].

### (c) Experimental period

At each of the six sites, each of the pair of feeders was designated as either the high-density or low-density feeder balancing the pre-experimental density evenly across the pair (i.e. in three sites the naturally lower density of the two was assigned to the ‘high density’ treatment, and the reverse for the other three sites). Of the birds (both great and blue tits) recorded at a given feeder site in the pre-experimental period, we randomly assigned 20% to the low-density feeder, with the remaining 80% assigned to the high-density feeder. Proportions of great and blue tits at low- and high-density sites were kept as consistent as possible (see the electronic supplementary material, table S1). These treatments were implemented by programming feeders such that the low-density feeder only allowed access to the birds on the relevant low-density list while the high-density feeder excluded birds on the low-density list from gaining access. Each feeder has a clear flap covering the feeding hole that can be programmed to open only when it reads the tag of an individual programmed to have access to that specific feeder, while recording the tags of all individuals that land on the perch. Birds can opt to scrounge at a feeder they were not given access to by attending the feeder once the flap has been opened by another bird, as it remains open for one to two seconds once triggered. Once programmed, feeders at each site were allowed to run for six weeks, with checks every two to three days to monitor feeder function, download data, and top-up sunflower seeds. Because great and blue tits spend substantial time processing seeds away from the feeder [50], birds exposed to the high- and low-density feeders had comparable access to resources, and thus the density manipulation is not expected to affect resource availability.

### (d) Statistical analysis

#### (i) Manipulation success

To assess whether the experimental manipulation had successfully increased the local density at one feeder within each site and decreased the local density at the other, we looked at temporal changes in the number of birds visiting feeders each day as well as the proportion of visits to a feeder site (i.e. a pair of feeders) that occurred at each feeder in the pair. To examine changes in local density, we calculated the number of individuals visiting per day and used this variable as the response in a generalized linear mixed effects model (GLMM) assuming Poisson errors. Similarly, to examine differences in visitation to the feeders, we calculated the proportion of daily visits that occurred at each feeder in each pair and used this as a response in a GLMM assuming binomial errors. In both cases, models contained the experimental treatment (high density or low density) in interaction with the period of the experiment (pre or during manipulation), and experimental day (here day was defined as the day within each period) as fixed effects, and we included feeder site as a random intercept to account for spatial heterogeneity in local density. We also used an auto-regressive error structure (ar1) to model temporal autocorrelation in daily measures within feeders. Both models were implemented using the ‘glmmTMB’ function in the ‘glmmTMB’ package [51].

We then used *post-hoc* comparisons between combinations of period and manipulation (using the ‘emmeans’ function in the ‘emmeans’ package [52]) to understand whether the experimental manipulation increased/decreased local density for the high-/low-density feeders respectively (i.e. within-feeder comparisons), and whether the experimental manipulation resulted in a difference in density between the assigned low- and high-density feeders before and during the experiment (i.e. between-feeder comparisons). Specifically, we carried out pairwise comparisons using Tukey-adjusted comparisons, with *p*-values adjusted for multiple comparisons.

#### (ii) Individual behaviour

To ensure that we were capturing variation in individual habitat choice decisions rather than variation in mortality or emigration outside of the woodland, we subsetted our data to only individuals that had (i) been recorded on a feeder four weeks after the start of the experiment, and/or (ii) had been captured as part of the long-term study between February and August 2021. We also only included birds that were recorded at least 100 times in the pre-experimental phase as prior to this point there was a clear relationship between the number of times a bird had been recorded on a feeder in the pre-experiment phase and their unweighted degree centrality. This restriction excluded 199 of 450 recorded birds. To ensure we were capturing true absences of birds at feeders, we also excluded days where either feeder in a pair recorded no activity, which is suggestive of a feeder malfunction.

#### (iii) Feeder usage

We first wanted to understand whether the density manipulation, an individual's social phenotype, as well as their interaction, predicted an individual's likelihood of continuing to exploit the feeder they were initially restricted to and/or their likelihood of choosing to switch to the alternative feeder despite not having access (i.e. scrounging). We did not analyse movements between experimental sites owing to their relative rarity (21 of 203 individuals moved between sites). To examine feeder usage, we used the feeder data to establish whether an individual had been recorded on their ‘home’ feeder (i.e. the feeder they were initially restricted to) and/or the alternative feeder (i.e. the other feeder in the pair) for each day of the experimental period. We then used each of these binary outcomes (recorded on the feeder = 1, not recorded = 0) as response variables in GLMMs with binomial errors and a logit link, again using the ‘glmmTMB’ function in‘glmmTMB’ [51].

For each of the two response variables (usage of home, usage of alternative feeder), we ran two models. The first included species (two-level factor), the day of the experiment, individual social phenotype (pre-experimental weighted degree), and the density manipulation an individual experienced at their home feeder (low density versus high density) as fixed effects. The second included all the fixed effects from the first model as well as an interaction between social phenotype and density manipulation to test for social phenotype-dependent responses to the density manipulation. In both models, we included experimental site and individual identity as random intercepts and used an auto-regressive error structure (ar1) to model temporal autocorrelation within individuals. We then used a likelihood ratio test to assess the support for the interaction term (‘lrtest’ function in the ‘lmtest’ package; v. 0.9–38 [53]).

#### (iv) Feeding behaviour

In addition to looking at effects of density and social phenotype on individual feeder choice, we wanted to examine whether birds altered their feeding behaviour in response to the density manipulations and whether any changes in feeding behaviour varied according to individual social phenotype. We characterized the feeding behaviour of individuals on their home feeder by looking at the daily mean visit length and the daily mean inter-visit interval (i.e. the length of time between visits). To calculate the mean length of feeder visits per individual per day, we first had to establish an interval that separated records in the same visit from those in another visit. To do this, we followed the methods of Milligan *et al*., [54] and used the minimum point between the peaks of the bimodal distribution of all inter-record intervals. The first peak occurring below two seconds is attributed to consecutive records when a bird remains on a perch while accessing the feeder, while the second peak (approx. 60 s) is attributed to the time necessary for a bird to process a seed in a nearby tree [54]. In our case, this interval corresponded to 22 s, and therefore, we grouped all records of an individual bird within 22 s of another record by that individual as the same visit.

We initially intended to also follow the methods of Milligan *et al*., [54] to distinguish visits within the same feeding bout from those occurring in different feeding bouts. However, when examining the distribution of all inter-visit intervals, our data did not follow as clearly a bimodal distribution as in their case. Therefore, we opted to simply average individual's daily inter-visit intervals regardless of length; unfortunately, when analysing this variable, we had problems with model convergence owing to its high skew. Thus, we excluded all inter-visit intervals that exceeded the 90th percentile (1382 s, or approx. 23 min) and re-calculated individual mean daily inter-visit intervals prior to the final analysis.

Models for visit lengths and inter-visit intervals had similar structures. The first model included species, experimental day, social phenotype and the density restriction as fixed effects, while the second also included an interaction between social phenotype and the density restriction an individual was exposed to. All models included feeder site and individual identity as random intercepts. For both visit length and inter-visit interval length, we assumed Gamma errors and a log link. Because we were examining feeding behaviour on the home feeder, and thus individuals only had visit length/inter-visit interval data when they were recorded on their home feeder, there were frequently gaps in the individual datasets. Consequently, there was little evidence for temporal autocorrelation within individuals, and we thus did not fit an autoregressive error term in these models.

All data manipulation and analyses were carried out using R v. 4.0.4. [55] and model performance was checked using the performance (v. 0.5.1 [56]) and DHARMa packages (v. 0.4–2; [57]).

## 3. Results

Of the 450 great and blue tits recorded in the pre-experimental phase across the six sites, we were left with 203 birds after restricting on pre-experimental observation frequency and survival to at least day 28 of the experiment. Of these 203 birds, 94 were blue tits and 109 were great tits. It is important to note that although we found some evidence for species differences in our analyses (see the electronic supplementary material, tables S4–S7), we do not discuss them further as our focus was on the role of social phenotype and social environment regardless of species.

### (a) Manipulation success

**Figure 2.**
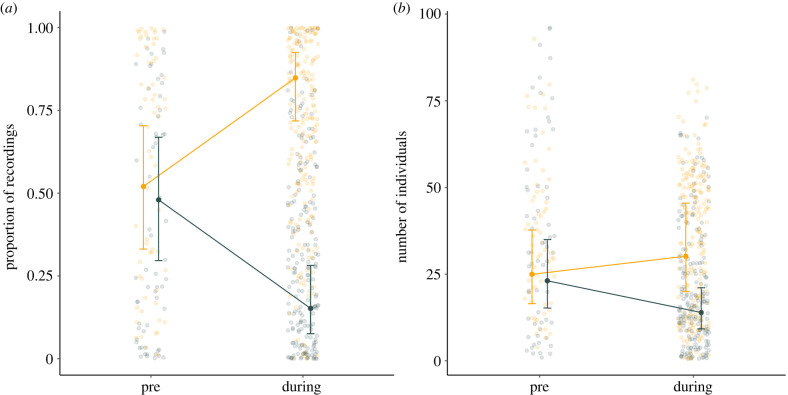
Both (*a*) the proportion of recordings and (*b*) the number of visiting birds varied between the low-density and high-density feeders during the experimental manipulation, but not in the pre-experimental phase, suggesting that the manipulation successfully altered the local social environment at each feeder. Shown are model estimates and standard errors overlaid on the raw data for each site before the experiment (pre) and during the experiment (during). Orange points and lines correspond to high-density feeders, while grey points and lines correspond to low-density feeders. (Online version in colour.)

Both local density and the proportion of feeder recordings occurring at feeders within experimental sites changed between pre-experiment and experimental periods. On average, the proportion of recordings at an experimental site increased from 52% to 85% between the pre-experimental period and the experimental period for the high-density feeder and decreased from 48% to 15% for the low-density feeder (figure 2*a*; see the electronic supplementary material, table S2 for model output). Similarly, the number of visiting birds increased by 30% from the pre-experimental period to the experimental period for the high-density feeder, while it decreased by 36% for the low-density feeder (figure 2*b*; see the electronic supplementary material, table S3 for model output). Thus, it was clear that the experimental manipulation successfully altered the local density experienced by birds restricted to a given feeder.

### (b) Feeder usage

**Figure 3.**
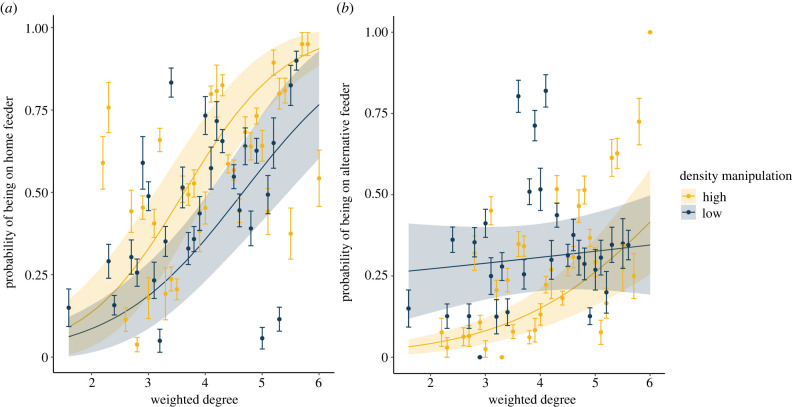
(*a*) We found no evidence for social phenotype-dependent responses to the density manipulations when examining the probability of birds being recorded on the feeder they were initially restricted to. (*b*) Birds restricted to the low-density manipulation were, on average, more likely to be recorded on the alternative feeder. However, for birds restricted to the high-density site, more social birds (higher weighted degree) were more likely to be recorded on the alternative feeder than less social ones. In both plots, points correspond to the raw data; however, we have combined individuals into bins (presenting means and standard errors within these bins) for clarity, with fitted relationships and associated standard error from the relevant mixed effects model overlaid. (Online version in colour.)

We tested the effect of the manipulation by analysing feeder choice within sites in relation to treatment and social phenotype. Birds restricted to the high-density feeder were 68% more likely to be recorded on the feeder they were initially restricted to than birds restricted to the low-density feeder (high density: est. = 63.9%, 95% confidence interval (CI) = 39.9–82.4; low density: est. = 38.1%, 95% CI = 18.1–63.1; see the electronic supplementary material, table S4 for best fit model output). In addition, birds that were more social (higher weighted degree) were also more likely to be recorded on their home feeder, irrespective of the treatment, on any given day (est. = 0.99, s.e. = 0.27, *p* < 0.001). We found little evidence for a significant interaction between the density restriction a bird was exposed to and their social phenotype on site choice (figure 3*a*; est. = 0.31, s.e. = 0.45, *p* = 0.48; *χ*^2^ = 0.29, *p* = 0.59), indicating that a bird's decision to remain in a particular social environment was not dependent on their social phenotype.

We also found effects of both the density manipulation and individual social phenotype when we examined the probability of birds being seen on the alternative feeder at the feeder site they were restricted to (i.e. the feeder they were not given access to). Birds restricted to the low-density feeder were twice as likely to be recorded at the alternative feeder on a given day (high density: est. = 15.4%, 95% CI = 6.6–32.0; low density: est. = 30.8%, 95% CI = 14.5–53.8; see the electronic supplementary material, table S5 for best fit model output). Similarly, we found moderate evidence that birds which were more social were also more likely to be recorded at the alternative feeder regardless of the manipulation they were exposed to (est. = 0.41, s.e. = 0.19, *p* = 0.03). By contrast to the probability of being recorded on their home feeder, we also found some evidence that the relationship between the probability of being recorded on the alternative feeder and social phenotype was dependent on the local density an individual experienced, with an increased probability for more social birds when restricted to the high-density feeder, but no relationship for birds restricted to the low-density feeder (figure 3*b*; est. = −0.61, s.e. = 0.30, *p* = 0.04; *χ*^2^ = 4.12, *p* = 0.04; see the electronic supplementary material, table S5 for best fit model output).

### (c) Feeding behaviour

**Figure 4.**
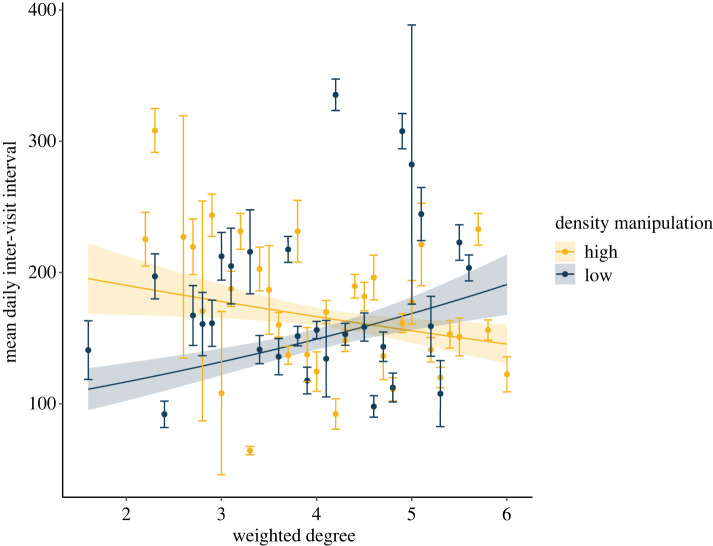
Birds with different social phenotypes responded differently to the density manipulations in terms of their inter-visit intervals, with less social birds (lower weighted degree) spending less time away from the feeder between visits when restricted to the low-density manipulation, while more social birds (higher weighted degree) spent less time between visits when restricted to the high-density manipulation. Shown are the raw data (means and standard errors) grouped for clarity, and fitted lines, with associated standard error, from the relevant mixed effects model overlaid. (Online version in colour.)

We further tested the effect of the experiment by analysing the temporal patterning of behaviour in relation to treatment and social phenotype. We found little evidence to suggest that the average length of feeder visits varied between the density treatments (est. = 0.04, s.e. = 0.05, *p* = 0.32; see the electronic supplementary material, table S6 for best fit model output), and weak evidence that it varied with individual social phenotype (est. = 0.04, s.e. = 0.02, *p* = 0.06). Furthermore, we found no evidence that responses to the experimental manipulation were dependent on an individual's social phenotype (est. = −0.009, s.e. = 0.05, *p* = 0.84). Similarly, when considering main effects only, we found no evidence for effects of density treatment (est. = −0.07, s.e. = 0.06, *p* = 0.24) or social phenotype (est. = 0.02, s.e. = 0.03, *p* = 0.53) on the average length of inter-visit intervals (see the electronic supplementary material, table S7 for best fit model output). However, in the case of inter-visit intervals, the relationship between social phenotype and inter-visit interval was dependent on the density treatment an individual was subject to (*χ*^2^ = 6.31, *p* = 0.01). For birds that were restricted to the low-density feeder, those that were more social spent longer away from the feeder between visits than less social individuals, while the opposite was true for birds restricted to the high-density feeder, with less social individuals having longer intervals (figure 4). This suggests that birds with different social phenotypes adjusted their feeding behaviour according to the social environment they were exposed to and is consistent with a hypothesis that asocial birds avoid high local densities.

## 4. Discussion

Habitat choice involves interactions with both con- and heterospecifics, whether positive or negative, and therefore is unavoidably tied to the social environment. Nevertheless, it has often been implicitly assumed that individuals will respond to the social environment in the same way. However, as research on animal personality has grown, there has been mounting appreciation of differences in social phenotype and thus social preferences [58–62]. This in turn raises the question of whether individuals with different social phenotypes may select for different social environments. Despite this, little work has investigated the importance of behavioural phenotype attributes in determining habitat choice behaviour, or explored the potential for individuals to choose to match their behavioural phenotype to their environment (i.e. matching habitat choice [13]) (but see [28]).

By experimentally exposing individuals with different social phenotypes to either high or low local density, and recording their subsequent foraging decisions, we showed that both the social environment and social phenotype predicted the decisions of birds to use both their home feeder and the alternative feeder at an experimental site. Our results suggest that more social birds were, on average, more likely to be recorded on both their home feeder and the alternative feeder on any given day. This result may suggest that more social birds have a higher probability of using both feeders to maintain or increase their social relationships. However, such a result could also arise if more social birds were also more explorative. A previous study in the Wytham population showed that fast-exploring individuals had a greater number of weak social associations than slow explorers [63]. However, estimated correlations between sociability and exploration have ranged from negative to positive in an array of animal systems [64–67]. Thus, further work is needed to better understand the tendency for sociability and exploration to be linked within and across species, and therefore the potential for such a behavioural syndrome to drive results such as ours.

Though we did find differences in feeder usage between less social and more social birds, we did not find evidence for matching habitat choice in terms of the likelihood of birds choosing to use high/low-density sites. Conversely, we did find evidence to suggest that individuals with different social phenotypes responded differently to the density manipulations in terms of their foraging behaviour at the site they were initially restricted to. This is suggestive of matching habitat choice at the level of feeder visits. Thus, our work indicates that personality traits may be important factors in explaining foraging decisions. Further, it also suggests that individuals may assess the match between their social phenotype and social environment and adjust their movement behaviour accordingly, but at a finer temporal scale. Even in the absence of spatial sorting, temporal clustering of this kind has the potential to lead to clustering of individuals with similar phenotypes, which may in turn facilitate the maintenance of phenotypic variation within populations [68] and even lead to evolutionary differentiation at small spatial scales [69].

Our finding that individuals with different social phenotypes adjusted their feeding behaviour differently when faced with the low- or high-density manipulation aligns both with previous work showing that social conditions can alter foraging behaviour [70–72] and that personality can affect an individual's foraging behaviour [73–75]. However, to our knowledge, our study is the first to demonstrate that individuals with different social phenotypes exhibit different foraging responses to the social environment. In our case, less social individuals seemingly chose to redirect their foraging effort elsewhere when restricted to the busy high-density feeders and consequently experienced longer inter-visit intervals when faced with this social environment. These birds may do this to avoid agonistic interactions in the vicinity of the feeder when competing for access to the feeder against a larger number of birds. By contrast, highly social individuals appear to approach a feeder more frequently when surrounded by others and therefore spend less time between visits when restricted to a high-density site. Thus, our findings build on other work suggesting that social and asocial individuals have different social preferences [37,58,76].

The differences in response to the experiment between birds with different social phenotypes may indicate different responses to competition. For example, less social birds may avoid busier environments, such as the high-density feeders in our experiment, to avoid agonistic interactions in the vicinity of the feeder and thereby increase their fitness. Thus, we suggest that competition may be the driver of matching habitat choice in our system, with birds of different social phenotype having differential preferences for high- and low-density habitats and adjusting their behaviour accordingly to increase their performance. However, it is possible that differential responses of birds with different social phenotypes to a given social environment may be driven instead by the exclusion of less competitive birds from otherwise preferred habitat [77]. Where competitive ability and sociability are correlated, then such a process may drive an apparent correlation between sociability and social environment. Thus, further work will be needed to establish whether the phenotype-dependent responses to social environment found here translated into differences in fitness and whether the apparent choices made by less social individuals are owing to exclusion from a location, or owing to less social individuals selecting locations with fewer competitors because they are less competitive.

Though our findings regarding inter-visit intervals may indicate that less social/more social birds choose to forage on resources away from the feeder itself when restricted to a high-density/low-density site, respectively (i.e. they select for different social environments), further study is required to understand the exact causes for these effects. For example, we could not quantify where birds spent their time when away from the feeder itself and therefore could not pinpoint characteristics of the environment they opted to forage in instead of at the feeder. To do so would require detailed information on the spatial locations of many hundreds of birds, data we are currently unable to collect. The rapid development of tracking technologies or automated experimental technologies may make more detailed studies of habitat choice in this and other systems feasible in the future, and such data are likely to facilitate the study of an array of additional questions surrounding the phenotypic and environmental determinants of variation in habitat choice.

We must also acknowledge that it is not possible to entirely exclude other mechanisms, such as habitat imprinting or genetic habitat preferences, that may have contributed to the differential responses of individuals with different social phenotypes to high- and low-density environments. We think habitat imprinting is unlikely to drive our results, as in the pre-experimental period the majority of birds (65%) used both feeders in a pair, suggesting that most birds were both familiar with and actively using both sites prior to the experiment. Excluding genetic preferences is more challenging; however, such an explanation also seems unlikely to have driven our results as it would necessitate a strong genetic component to both social phenotype and habitat preference, as well as a genetic correlation between the two [78]. Nevertheless, this serves to highlight the need for further work exploring the genetic underpinnings of habitat preferences. Such knowledge will be critical both to better understand the consequences of habitat choice behaviour but also to enable improved estimates of trait heritabilities and thereby predictions of evolutionary change.

Our results raise the question as to why we found that individuals with different social phenotypes responded differently to the high/low density manipulations in terms of their foraging behaviour but not in their likelihood of scrounging at the alternative feeder. One potential cause is birds opting to maintain social bonds established prior to the manipulation, and previous work in this system and others has shown that individuals trade-off access to food against maintaining social relationships ([41]; see also [79,80]). For example, birds in this system have been shown to scrounge at a feeder they do not have access to in order to maintain their relationship with their mated partner. Such a tendency to prioritize social relationships over access to food could explain why birds restricted to the low-density manipulation were more likely to attempt to scrounge at the alternative feeder. Birds allocated to the low-density treatment were on average three times more likely to have been separated from the bird they were most closely associated with in the pre-experimental period than birds restricted to the high-density feeder and thus may have opted to scrounge at the alternative feeder to maintain their pre-experimental bonds (see the electronic supplementary material, table S8 for details). Nevertheless, the fact we found phenotype-dependent responses at the visit level, but not at the daily scale adds to the considerable literature demonstrating the scale-dependence of habitat selection/foraging decisions [81–83].

In conclusion, we show that both social phenotype and social environment affect individual habitat choice and foraging decisions. Furthermore, we found evidence that birds with different social phenotypes adjusted their foraging behaviour differently to exposure to high/low density. In doing so, our work demonstrates the importance of differences in social phenotype for determining foraging decisions, as well as indicating further support for the idea of personality matching habitat choice, though in this case focused on sociability rather than boldness [28]. Further work will be needed to establish the general importance of social phenotype in determining movement decisions, as well as how behavioural traits interact with other aspects of the phenotype to determine habitat choice decisions and foraging behaviour. Furthermore, although in this study our density manipulations involved changing the exposure of individuals to both con- and hetero-specifics, it is possible that individuals differ in their preferences for individuals of the same versus different species. Indeed, a study in the Wytham tit population has demonstrated that individual birds differ consistently in the degree to which they interact with heterospecifics [40]. Thus, future work should focus not only on differences between individuals in their overall sociability, but also whether individuals may differ in their sociability towards individuals of the same versus different species. Continued work exploring the potential for matching habitat choice behaviour has the potential to shed light on the consequences of habitat choice for phenotypic structure within populations and thus for population and evolutionary dynamics [13,20,23].

## Data Availability

The data and code supporting the findings in this study are openly available from the Dryad Digital Repository: https://doi.org/10.5061/dryad.crjdfn379 [84]. Information is also provided in the electronic supplementary material [85].
